# Improved multi-level protein–protein interaction prediction with semantic-based regularization

**DOI:** 10.1186/1471-2105-15-103

**Published:** 2014-04-12

**Authors:** Claudio Saccà, Stefano Teso, Michelangelo Diligenti, Andrea Passerini

**Affiliations:** 1 Dipartimento di Ingegneria dell’Informazione e Scienze Matematiche, University of Siena, Siena, Italy; 2 Dipartimento di Ingegneria e Scienza dell’Informazione, University of Trento, Trento, Italy

## Abstract

**Background:**

Protein–protein interactions can be seen as a hierarchical process occurring at three related levels: *proteins* bind by means of specific *domains*, which in turn form interfaces through patches of *residues*. Detailed knowledge about which domains and residues are involved in a given interaction has extensive applications to biology, including better understanding of the binding process and more efficient drug/enzyme design. Alas, most current interaction prediction methods do not identify which parts of a protein actually instantiate an interaction. Furthermore, they also fail to leverage the hierarchical nature of the problem, ignoring otherwise useful information available at the lower levels; when they do, they do not generate predictions that are guaranteed to be consistent between levels.

**Results:**

Inspired by earlier ideas of Yip *et al.* (BMC Bioinformatics 10:241, 2009), in the present paper we view the problem as a multi-level learning task, with one task per level (proteins, domains and residues), and propose a machine learning method that collectively infers the binding state of all object pairs. Our method is based on Semantic Based Regularization (SBR), a flexible and theoretically sound machine learning framework that uses First Order Logic constraints to tie the learning tasks together. We introduce a set of biologically motivated rules that enforce consistent predictions between the hierarchy levels.

**Conclusions:**

We study the empirical performance of our method using a standard validation procedure, and compare its performance against the only other existing multi-level prediction technique. We present results showing that our method substantially outperforms the competitor in several experimental settings, indicating that exploiting the hierarchical nature of the problem can lead to better predictions. In addition, our method is also guaranteed to produce interactions that are consistent with respect to the protein–domain–residue hierarchy.

## Background

Physical interactions between proteins are the workhorse of cell life and development [1], and play an extremely important role both in the mechanisms of disease [2] and in the design of new drugs [3]. In recent years, there has been enormous interest in reverse engineering the protein–protein interaction (PPI) networks of several species, particularly due to the availability of high-throughput experimental techniques, leading to an abundance of large databases on all aspects of PPIs [4].

Notwithstanding the increased availability of interaction data, the natural question of whether two arbitrary proteins interact, and why, is still open. The growing literature on protein interaction prediction [4-6] is symptomatic of the gap separating the amount of available data and the effective size of the interaction network [7]. The present paper is a contribution towards filling this gap.

**Figure 1:**
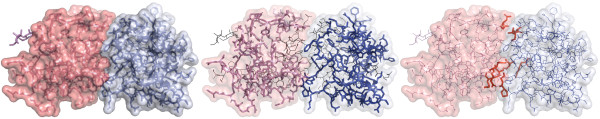
**The protein–domain–residue hierarchy.** Two bound proteins and their interacting domains and residues, captured in PDB complex 4IOP. The proteins are a Killer cell lectin-like receptor (in violet) and its partner, a C-type lectin domain protein (in blue). (Left) Interaction as visible from the contact surface. (Center) The two C-type lectin domains instantiating the interaction. (Right) Effectively interacting residues in red.

Our work is based on the observation that physical interactions can be viewed at three levels of detail. At a higher level, two proteins interact to perform some function within a biological pathway (e.g. metabolism, signaling, regulation, *etc*.) [8]. At a lower level, the same interaction occurs between a pair of specific *domains* appearing in the proteins; the types of the domains involved characterize the functional semantics of the interaction [9]. At the lowest level, the interaction is instantiated by the binding of a pair of protein *interfaces*, patches of solvent accessible residues with compatible shapes and chemical properties [10]. The low-level features of the binding sites determine whether the interaction is transient or permanent, whether two proteins compete for interaction with a third one, *etc.* Figure 1 illustrates the multi-level mechanisms with an example taken from the PDB.

Despite the significance of low-level details in elucidating the mechanics of protein–protein interactions, most of the current experimental data comes from high-throughput screening techniques, such as yeast two-hybrid (Y2H) assays [11]. These techniques do *not* provide information on domain- or residue-level interactions, which require solving the three-dimensional structure of each protein-protein complex, an expensive and time consuming task addressed by X-Ray crystallography, NMR, or electron microscopy techniques [12]. As a consequence, protein–protein interaction data is under-characterized at the domain and residue levels: the current databases are relatively lacking when compared to the magnitude of the existing body of data about protein-level interactions [13]. At the time of writing, the PDB hosts 84,418 structures, but merely 4,210 resolved complexes (according to http://www.rcsb.org/pdb/statistics/holdings.do, retrieved on 2013/06/20). The latter cover only a tiny fraction of the interactions stored in databases such as BioGRID and MIPS.

From a purely biological perspective, predictions at different levels have several important applications. The network topology and individual features of protein interactions are an essential component of a wide range of biological tasks: inferring protein function [14] and localization [15], reconstructing signal and metabolic pathways [16], discovering candidate targets for drug development [2]. Finer granularity predictions at the domain level allow to discover affinities between domain types that can be carried over to other proteins [17,18]; domain–domain networks have also been assessed as being typically more reliable than their protein counterparts [13]. Finally, residue-level predictions, i.e., interface recognition, enable the detailed study of the principles of protein interactions, and are crucial for tasks such as rational drug design [3], metabolic reconstruction and engineering [19], and identification of hot-spots [20] in the absence of structure information.

Given the usefulness of knowing the details of protein–protein interactions at diverse levels of detail, and based on earlier ideas of Yip *et al.*[21], in this paper we address the problem of collectively predicting the binding state of all proteins, domains, and residues in a network. We call this task the *multi-level protein interaction prediction* problem (MLPIP for short).

From a computational point of view, the most important feature of the multi-level prediction problem is its inherently relational nature. Proteins, domains and residues are organized in a hierarchy, which dictates constraints on the binding state of pairs of objects at the different levels, as follows. On the one hand, whenever two proteins are bound, at least two of their domains must also be bound, and, similarly, there must be residues in the two domains that form an interface. On the other hand, if no residues of the two proteins interact, neither do their domains, nor the proteins themselves. In other words, predictions at different levels must be *consistent*.

In this paper we cast the multi-level prediction problem as a statistical-relational learning task, leveraging the latest developments in the field. Our prediction method is based on Semantic Based Regularization [22], an elegant semi-supervised prediction framework that caters both the effectiveness of kernel machines and the expressivity of First Order Logic (FOL). The constraints described above are encoded as FOL rules, which are used to enforce consistent predictions at all levels of the interaction hierarchy. By computing multi-level predictions, our method can not only infer which protein pairs are likely to interact, but also provide details about how the interactions take place. Our empirical evaluation shows the effectiveness of this constraint-based approach in boosting predictive performance, achieving substantial improvements over both an unconstrained baseline and the only existing alternative MLPIP method [21].

### Problem definition

PPI networks are most naturally formalized as graphs, where nodes represent proteins and edges represent interactions. Given a set of features describing the properties of the proteins in the network (e.g. primary structure, localization, tertiary structure —when available—, *etc.*), inferring the PPI network topology amounts to determining those pairs of proteins that are likely to interact. This task is often cast as a pairwise classification problem, where a binary classifier takes as input a pair of proteins (or rather their feature-based representations) and predicts whether they interact or not. Standard binary classification methods, such as Support Vector Machines [23], can be used to implement the pairwise classifier. In this setting, the interaction depends only on the features of the two incident nodes, and is independent of all other nodes. Interactions between domains or residues can be predicted similarly.

The most straightforward way to address the MLPIP problem is to cast the three interaction prediction problems, for proteins, domains and residues respectively, as independent pairwise classification tasks. However, as previously discussed, these problems are clearly strongly related: two proteins interact via one or more domains, which in turn contain patches of residues that constitute the interaction surface. Ignoring these relationships can lead to heavily suboptimal, inconsistent predictions, where, e.g. two proteins are predicted to interact but none of their domains are predicted to be involved in this interaction. Making these relationships explicit and forcing predictors to satisfy consistency constraints is the key contribution of this work. In the machine learning community, this kind of scenario characterized by multiple related prediction tasks is usually cast as a statistical-relational learning problem [24,25], where the goal is to *collectively* classify the state of all objects of interest, taking into account the relations existing between them. The solution we adopt is grounded in this learning framework.

### Overview of the proposed method

**Figure 2:**
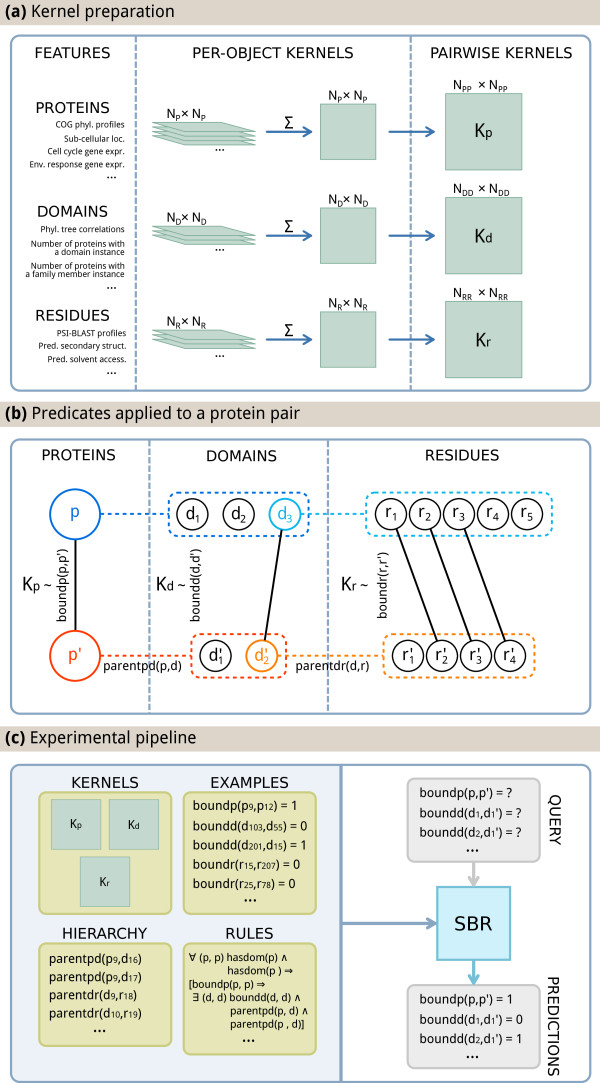
**Visualization of the proposed method.** Visualization of the proposed method. **(a)** Kernel preparation at the three levels. A kernel is derived for each input feature (Left); the resulting matrices are summed up to obtain a per-object kernel (Middle), which is transformed into a pairwise kernel using Eq 1. Here *N*_*p*_ (*N*_*d*_, *N*_*r*_) is the number of individual proteins (respectively domains, residues) in the level, while *N*_*pp*_ (*N*_*dd*_, *N*_*rr*_) is the number of protein (respectively domain, residue) interactions in the dataset. **(b)** Instantiation of all predicates (Table 1) over a pair of proteins p’ and p’ and their parts. Circles represent proteins, domains and residues. Dotted lines indicate a parent-child relationship between objects, representing the parentpd and parentdr predicates. Solid lines link pairs of bound objects, i.e. objects for which the boundp, boundd or boundr predicates are true. **(c)** Visualization of the experimental pipeline. Given the pairwise kernels, the set of rules (Table 2), a set of example interactions, and a description of the protein-domain-residue hierarchy, SBR finds a prediction for the query predicates consistent with the rules.

In this paper we propose solving the multi-level prediction problem adapting a state-of-the-art statistical-relational learning framework, namely Semantic Based Regularization (SBR) [22]. SBR ties multiple learning tasks, which are themselves addressed by kernel machines, using constraints expressing First Order Logic knowledge. In the following we give an overview of the SBR framework, also pictured in Figure 2; see Methods for further details.

**Table 1:** Predicates

**Target predicates**	
boundp(p,p’)	true iff the protein pair (p,p’) is bound
boundd(d,d’)	true iff the domain pair (d,d’) is bound
boundr(r,r’)	true iff the residue pair (r,r’) is bound
**Given predicates**	
parentpd(p,d)	true iff protein p is parent of domain d
parentdr(d,r)	true iff domain d is parent of residue r
parentpr(p,r)	true iff protein p is parent of residue r
hasdom(p)	true iff protein p has at least one domain
hasres(d)	true iff domain d has at least one residue

List of predicates used by SBR.

**Table 2:** Rules

**Name**	**Definition**
P →D	∀(p,p’)hasdom(p) ∧hasdom(p’) ⇒
	boundp(p,p’) ⇒ ∃(d,d’)boundd(d,d’) ∧parentpd(p,d) ∧parentpd(p’,d’)
D →P	∀(p,p’) ∃(d,d’)
	boundd(d,d’) ∧parentpd(p,d) ∧parentpd(p’,d’) ⇒boundp(p,p’)
D →R	∀(d,d’)hasres(d) ∧hasres(d’) ⇒
	boundd(d,d’) ⇒ ∃_*n*_(r,r’)boundr(r,r’) ∧parentdr(d,r) ∧parentdr(d’,r’)
R →D	∀(d,d’) ∃(r,r’)
	boundr(r,r’) ∧parentdr(d,r) ∧parentdr(d’,r’) ⇒boundd(d,d’)
P →R	Same as D →R, with proteins in place of domains
R →P	Same as R →D, with proteins in place of domains

List of FOL constraints used by SBR.

Let  be a set of objects. In most scenarios, objects are *typed*, so that objects of the same type can be considered as belonging to the same group. In our setting, object types are proteins, domains and residues, with corresponding sets $X_{P},X_{D}$ and $X_{R}$ respectively. Predicates represent properties of objects or relationships between them. Depending on the scenario, some predicates are always known (called *given* predicates), some other are known only for a subset of the objects, and their value should be predicted when unknown (*query* or *target* predicates). The parentpd(p,d) predicate, for instance, specifies that domain $\text{d}\in X_{D}$ is part of protein $\text{p}\in X_{P}$, i.e. the predicate is true for all (p,d) pairs for which d is a domain of p, and false otherwise. The value of this predicate is known for all objects in our domain; note that there indeed are many proteins whose domains are unknown, but in this case there is no corresponding domain object in our data). The boundp(p,p’) predicate specifies whether two proteins p and p’ are interacting. This is one of the target predicates, whose truth value should be predicted for novel protein-protein pairs. Similar predicates are defined for domain and residue level bindings. Target predicates are modelled as binary classifiers, i.e. functions trained to predict the truth value of the predicate. Relationships between predicates can be introduced in order to enforce constraints known to hold in the domain. SBR allows to exploit the full power of First Order Logic in doing this. As a matter of example, the notion that two interacting proteins should have at least one interacting domain can be modelled as (see Methods for details on First Order Logic notation):

$$\begin{array}{ll} \forall \text{(p,p’) boundp(p,p’)}\Rightarrow \; & \exists \;\text{(d,d’)}\;\text{boundd(d,’d)}\;\wedge \\ \text{parentpd(p,d)}\;\wedge \\ \text{parentpd(p’,d’)} \end{array}$$

Each binary classifier is implemented in the SBR framework as a kernel machine [26]. The key component of kernel machines is the kernel function, which measures the similarity between objects in terms of their representations. A protein, for instance, can be represented as the sequence of its residues, plus additional information as its subcellular localization and/or its phylogenetic profile. Having the same subcellular localization, for instance, should increase the similarity between two proteins, as having a similar amino acid composition. Designing appropriate kernels is a crucial component of a successful predictor. A kernel machine is a function which predicts a certain property of an object *x* in terms of a weighted sum of similarities to other objects for which the property is known, i.e.:

$$f(x)=\underset{i}{\sum }w_{i}K(x,x_{i})$$

A kernel machine could for instance predict whether a protein is an enzyme or not (binary classification), in terms of weighted similarity to other proteins. Being similar to an enzyme *x*_*i*_ will drive the prediction towards the positive (enzyme) class (positive weight *w*_*i*_), while being similar to a non-enzyme *x*_*j*_ will drive the prediction towards the opposite class (negative weight *w*_*j*_).

In the interaction prediction setting, target predicates actually predict properties of *pairs* of objects (proteins, domains or residues). We thus employ a pairwise kernel machine classifier to model the target predicate:

$$f(x,x^{\prime })=\underset{i}{\sum }w_{i}K((x,x^{\prime }),(x_{i},x_{i}^{\prime }))$$

Here the kernel function measures the similarity between two pairs of objects, so that, e.g. two proteins will be predicted as interacting if they are similar to protein pairs which are known to interact, and dissimilar from pairs known to not interact.

Given a kernel between objects *K*(*x*,*x*^′^), it is possible to construct a pairwise kernel by means of a the following transformation [27]:

(1) $$\begin{array}{ll} K((x_{i},x_{j}),(x_{k},x_{l}))= & K(x_{i},x_{k})\cdot K(x_{i},x_{l})+\\ & K(x_{j},x_{k})\cdot K(x_{j},x_{l}) \end{array}$$

This transformation guarantees that, if the input function *K* is a valid kernel, so is the resulting pairwise function.

As already explained, in SBR each target predicate is implemented as a kernel machine, and the state of a predicate for an uncharacterized pair of proteins can be inferred by querying the machine. Positive predictions correspond to true predicates, i.e. bound protein pairs, and negative predictions to false ones. The confidence of the kernel machine, also called *margin*, embodies the confidence in the state of the predicate, that is, how strongly two proteins are believed to interact (or not).

Given the output of the kernel machines for all target predicates, SBR uses the First Order Logic rules to condition the state of the correlated predicates. It does so by first translating the FOL rules into continuous constraints, which we discuss more thoroughly in Methods. The variables coming into play into the continuous constraints are the confidences of all target predicates (and the state of all given predicates) appearing in the equivalent FOL constraint. The amount of violation is reflected by the value of the continuous constraints: if the predicted predicates satisfy a FOL rule, the corresponding constraint will have a value equal to 1; on the other hand, the closer the constraint value to zero, the more the FOL rule is violated.

SBR computes a solution to the inference problem, i.e. deciding the truth value of all target predicates, that maximizes both the confidence of individual predicates and the amount of satisfaction of all constraints. Informally, the optimal assignment to all predicates, i.e. the binding state of protein, domain and residue pairs, *y*^∗^, is a solution to the following optimization problem:

$$y^{*}=\arg\;\underset{y}{\max}\;\text{consist}\;(y,f)+\;\text{consist}\;(y,\text{KB})$$

where the first term accounts for consistency between inferred truth values and confidence of the individual predictions, and the second incorporates information on the degree of satisfaction of the constraints build from the FOL knowledge. Contrarily to standard kernel methods, this optimization problem is non-convex. This is commonly the case for complex statistical-relational learning tasks [24], and implies that we are restricted to finding local optima. SBR employs a two-stage learning process to make training effective even in presence of local optima. In particular, the first stage of SBR learning takes into account the fitting of the individual predictions to the supervised data. This learning task is convex and can be efficiently solved. The solution found in the first stage is used as starting point for a second stage, where the FOL knowledge is also considered. This optimization strategy has been experimentally proved to find high-quality solutions without adding the computational burden of other non-convex optimization techniques [22].

SBR is a semi-supervised method [28], meaning that the set of target proteins is given beforehand and can be exploited during the learning stage to fine-tune the model. Semi-supervised learning is known to enhance the prediction ability when appropriately used [29], and can be applied very naturally to PPI prediction, as the full set of proteins is always known.

To summarize, at each level the state of an uncharacterized pair of objects, e.g. proteins *p* and *p*^′^, is mainly inferred by the similarity of the pair (*p*,*p*^′^) to other pairs that are known to interact or not, through the pairwise kernel function *K* and the learned weights ***w***. Thus the kernel allows to propagate information *horizontally* within the same level. At the same time, the FOL constraints allow to propagate information *vertically* between the levels, by keeping the interaction pattern along the protein-domain-residue hierarchy consistent.

### Modeling multi-level interactions

As already explained, we use two distinct kinds of predicates: given predicates and target predicates. Given predicates encode *a priori* knowledge about the problem, in our case the structure of the multi-level object hierarchy. In particular, given a protein p and a domain d, the parentpd(p,d) predicate is true if and only if domain d occurs in protein p; the parentdr predicate is the analogous for domains and residues. This simple representation suffices to encode the whole protein–domain–residue hierarchy. To simplify the notation, we also introduce the hasdom(p) predicate to encode the fact that protein p has at least one domain. More formally:

hasdom(p):=∃dparentpd(p,d)

The hasdom predicate can be computed directly by SBR using the above definition; we instead pre-compute its value for all protein pairs for run-time efficiency.

The boundp(p,p’)*target* predicate models the binding state of two distinct proteins. Its state is known for certain protein pairs, i.e. those in the training set, and our goal is to predict its state on the remaining ones. The boundd(d,d’) predicate plays the same role for domains. For a complete list of predicates, see Table 1. For a visualization of the predicates instantiated over a protein pair, see Figure 2-b.

In what follows we describe how to design inter-level FOL constraints to properly enforce consistency between predictions at different levels. We focus on modeling the constraints tying proteins and domains; it is easy to see that the ones between domains and residues can be modelled similarly (with one peculiar exception that will be pointed out later). Table 2 reports the complete list of rules.

Inter-level constraints can be seen as propagating information from the upper layer to the lower one and in the opposite direction. To model this mechanism, we use two distinct constraints: the P →D rule and the D →P rule. A simplified version of the P →D rule is:

$$\begin{array}{ll} \forall \;\text{(p,p’)}\;\text{boundp(p,p’)}\Rightarrow & \;\;\exists \text{(d,d’)}\;\text{boundd(d,d’)}\wedge \\ \;\;\text{parentpd(p,d)}\;\wedge \\ \;\;\text{parentpd(p’,d’)} \end{array}$$

Intuitively, the rule means that whenever two proteins are bound (and therefore the left-hand side (LHS) of the implication is true) then there must be *at least one* pair of child domains that are bound (the right-hand side (RHS) is true). In classical First Order Logic the rule would require that, whenever none of the child domains is bound (the RHS is false), then the parent proteins must not be bound (the LHS is false).

Note that, in the above formulation, the rule is applied indiscriminately to *all* protein pairs, even to those that have no known child domains in the considered dataset. Therefore, the rule can be reformulated in order to enforce it only for those protein pairs that do in fact have child domains, using the hasdom predicate, as follows:

$$\begin{array}{ll} \forall \;\text{(p,p’)}\;\text{hasdom(p)}\wedge & \;\text{hasdom(p’)}\Rightarrow \\ \;\left(\text{boundp(p,p’)}\right)\Rightarrow & \;\exists \text{(d,d’)}\;\text{boundd(d,d’)}\wedge \\ & \;\text{parentpd(p,d)}\wedge \\ & \;\;\;\;\left(\text{parentpd(p’,d’)}\right) \end{array}$$

This is the complete P →D rule. The left-hand side is always false for proteins without domains, making the rule always satisfied in this case (effectively disabling the effect of the rule on the learning process). We define the complementary D →P rule as follows:

$$\begin{array}{ll} \forall \text{(p,p’)} & \left(\exists \text{(d,d’)}\;\text{boundd(d,d’)}\;\wedge \right)\\ & \text{parentpd(p,d)}\;\wedge \\ & \text{parentpd(p’,d’)}\\ & \left(\Rightarrow \;\text{boundp(p,p’)}\right) \end{array}$$

This rule is applied to all protein pairs, demanding that if there is a pair of bound children domains then the proteins must be bound too, and vice versa that if the parent proteins are unbound so are the domains. The P →D and D →P rules could be merged into a single equivalent rule using the double implication (⇔). However, the rules have been considered separately to keep their effects on the results separated and easier to analyze.

To simulate the unidirectional information propagation between levels, as done by Yip *et al.*[21] (see Related work), we modified how SBR converts logic implications by using the t-norm residuum, which states that a logic implication is true if the RHS is at least as true as the LHS. This modification also removes a bias in the translation of the implication that was affecting the original formulation of SBR, whose effect is to often move the LHS toward the false value. See Methods for details.

The constraints for domains and residues can be similarly defined with one important exception. The P →D rule described above (correctly) requires *at least one* domain couple to be bound for each interacting protein pair. However, when two domains are bound, the interaction interface involves more than one residue pair: for instance, binding sites collected in the protein–protein docking benchmark version 3.0 [30] consist of 25 residues on average [31]. We integrate this observation in the D →R rule using the *n*-existential operator ∃_*n*_ in place of the regular existential (see Table 2 for the complete formulation), so that whenever two domains are bound, *at leastn* pairs of their residues must be bound. Since interfaces in the employed dataset are typically 5 residues long, *n*=5 has been used in the experiments. Our results demonstrate that this seemingly small modification has a rather extensive impact on the prediction of domain and residue level interactions.

### Related work

In this section we briefly summarize previous PPI interaction prediction approaches using methods that are most closely related to the present paper: kernel methods, semi-supervised methods, and logic-based methods. For a broader exposition of interaction prediction methods, please refer to one of the several surveys on the subject [4,6,9,32].

The earliest attempt to employ kernel methods [26] for PPI prediction is the work of Bock *et al.*[33], which casts interaction prediction as pairwise classification, using amino-acid composition and physico-chemical properties alone. Ben-Hur *et al.*[27] extended the previous work by applying pairwise kernels and combining multiple data sources (primary sequence, Pfam domains, Gene Ontology annotations and interactions between orthologues). Successive publications focused primarily on aggregating more diverse sources, including phylogenetic profiles, genetic interactions, and subcellular localization and function [6]. Kernel machines have also been applied to the prediction of binding sites from sequence, as summarized in [10]. The appeal of supervised kernel methods is that they provide a proved and theoretically grounded set of techniques that can easily integrate various information sources, and can naturally handle noise in the data. However, they have two inherent limitations: (i) the binding state of two proteins is inferred independently from the state of all other proteins, and (ii) due to their supervised nature, they do not take advantage of unsupervised data, which is very abundant in the biological network setting.

Semi-supervised learning (SSL) techniques [28,29] attempt to solve these issues. In the SSL setting the set of target proteins is known in advance, meaning that the learning algorithm has access to their distribution in feature space. This way the inference task can be simplified by introducing unsupervised constraints that assign the same label to proteins that are, e.g., close enough in feature space, or linked in the interaction network, instantiating a form of information propagation. There are several works in the PPI literature that embed the known network topology using SSL constraints. Qi *et al.*[34] employ SSL methods to the special case of viral-host protein interactions, where supervised examples are extremely scarce. Using similar methods, You *et al.*[35] attempt to detect spurious interactions in a known network by projecting it on a low-dimensional manifold. Other studies [36,37] applied SSL techniques to the closely related problems of gene–protein and drug–protein interaction prediction. Despite the ability of SSL to integrate topology information, no study so far has applied it to highly relational problems such as the MLPIP.

An alternative strategy for interaction prediction is Inductive Logic Programming (ILP) [38], a group of logic-based formalisms that extract rules explaining the likely underlying causes of interactions. ILP methods were studied in the work of Tran *et al.*[39] using a large number of features: SWISS-PROT keywords and enzyme properties, Gene Ontology functional annotations, gene expression, cell cycle and subcellular localization. Further advances in this direction, with a special focus on using domain information, can be found in [17,18]. The advantage of ILP methods over purely statistical methods is that they are inherently able to deal with relational information, making them ideal candidates for solving the MLPIP problem. Alas, contrary to kernel methods, they tend to be very susceptible to noise, which is a very prominent feature of interaction dataset, and are less effective in exploiting complex feature representations, e.g. involving highly non-linear interactions between continuous features.

Recently, some works highlighted the importance of the multi-level nature of protein–protein interactions. Gonzalez *et al.*[40] propose a method to infer the residue contact matrix from a known set of protein interactions using SVMs; on the contrary, our goal is to predict the interactions concurrently at all levels of the hierarchy. Another study [13] highlights the relevance of domain-level interactions, and the unfortunate lack of details thereof, and formulates a method to reinterpret a known PPI network in terms of its constituent domain interactions; the present work has a different focus and a more general scope.

Most relevant to this paper is the work of Yip *et al.*[21], where the authors propose a procedure to solve the MLPIP problem based on a mixture of different techniques. The idea is to decompose the problem as a sequence of three prediction tasks, which are solved iteratively. Given an arbitrary order of the three levels (e.g. proteins first, then domains, then residues), their procedure involves computing putative interactions in the first level (in this case proteins), then using the most confident predictions as novel training examples at the following level (i.e., domains). The procedure is repeated until a termination criterion is met.

Intra-level predictions are obtained with Support Vector Regression (SVR) [41]. In particular, each object has an associated SVR machine that models its propensity to bind any other object in the same level. The extrapolated values act as confidences for the predictions themselves. The mechanism for translating the most confident predictions at one level into training examples for the next level depends on the relative position of the two levels in the hierarchy. Downward propagation (e.g. from proteins to domains) simply associates to each novel example the same confidence as the parent prediction: in other words, if two proteins are predicted as bound with high confidence, all their domains will be considered bound with the same confidence. Upward propagation (e.g. from domains to proteins) is a bit more involved: the confidence assigned to the novel example (protein) is a noisy-OR combination of confidences for all the involved child objects (domains).

While this method has been shown to work reasonably well, it is afflicted by several flaws. First of all, while the iterative procedure is grounded in co-training [42], the specific choice of components is not as theoretically sound. For instance, the authors apply regression techniques on a classification task, which may lead to sub-optimal results. The inter-level example propagation mechanisms are *ad hoc*, do not exploit all the information at each level (only the most confident predictions are propagated), and are designed to merely propagate information between levels, not to enforce consistency on the predictions. In particular, the downward propagation rule is rather arbitrary: it is not clear why *all* domains of bound proteins should be themselves bound with the same confidence. Finally, these rules, which are intimately tied to the specific implementation, are not defined using a formal language, and are therefore difficult to extend. For instance, it would be difficult to implement in said framework something similar to an *n*-existential propagation rule, which is extremely useful for dealing with residue interactions.

Semantic Based Regularization seems to have many obvious advantages in this context. A first advantage is that it decouples the implementation of the functions from how consistency among levels is defined. Indeed, consistency is implemented via a set of constraints, which are applied over the output of the predictors. However, there is no limitation to which kind of predictors are used. For example, we used kernel machines as basic machinery for implementing the predictor, where different state-of-the-art kernels can be used at the single levels, while still be able to define a single optimization problem.

Furthermore, SBR allows to natively propagate the predictions of one level to the other levels. Since the predictions and not the supervisions are propagated, SBR accuracy can get advantage of the abundant unsupervised data. The availability of an efficient implementation of the *n*-existential quantifier is also a crucial advantage: if two proteins or domains are interacting, a small set of residues must be interacting as well. SBR does not simply propagate a generic prior to all the residues for a protein or domain, which could decrease accuracy of the reductions for the negative supervisions. SBR instead performs a search process in order to select a subset of residue candidates, where to enforce the interaction. As shown in the experimental results, this greatly improves residue prediction accuracy. Finally, the circular dependencies that make learning difficult are dealt in the context of a general and well defined framework, which implements various heuristics to make training effective.

## Results and discussion

### Dataset

In this work we use the dataset of Yip *et al.*[21], described here for completeness. The dataset represents proteins, domains and residues using features gathered from a variety of different sources: 

• Protein features include phylogenetic profiles derived from COG, subcellular localization, cell cycle and environmental response gene expression; protein-pair features were extracted from Y2H and TAP-MS data. The gold standard of positive interactions was constructed by aggregating experimentally verified or structurally determined interactions taken from MIPS, DIP, and iPfam.

• At the domain level, the dataset includes both features for domain families and for domain instances based on frequencies of domains within one or more species and phylogenetic correlations of Pfam alignments. The gold standard of positive interactions was built from 3D structures of complexed proteins taken from PDB.

• Residue features consist of sequence-based properties, namely charge complementarity, Psi-Blast [43] profiles, predicted secondary structure, and predicted solvent accessibility.

Kernels computed from the individual features were combined additively into a single kernel function for each level, and then transformed into pairwise kernels using Equation (1); the resulting functions were used as inputs to SBR. A visualization of the process can be found in Figure 2.

This procedure yields a dataset of 1681 proteins, 2389 domains, and 3035 residues, with a gold standard of 3201 positive (interacting) protein pairs, 422 domain pairs, and 2000 residue pairs. Since interaction experiments can not determine which pairs do *not* interact, the gold standard of negative pairs is built by randomly sampling, at each level, a number of pairs that are not known to interact (*i.e.* not positive). This is a common approach to negative labeling in the PPI prediction literature [44]. To keep the dataset balanced, the number of sampled negative pairs is identical to the number of objects in the gold standard of positives. For more details on the dataset preparation, please refer to [21]. We further refined the dataset by running CD-HIT [45] with a 20% sequence similarity threshold, identifying 23 redundant proteins. These proteins were not used when comparing the method performances.

Turning our attention to the resulting dataset, we note that most of the supervision is located at the protein level: out of all possible interactions between pairs of proteins, which are (1681×1680)/2, 0.226% are known (either positive or negative). On the contrary, the other levels hold much less information: only 0.042% of all possible residue pairs, and 0.014% of all possible domain pairs, are in the dataset. The low number of residue pairs is due to i) different requirements for experimentally determining the interactions at the three levels, i.e. whether the structure is available; and ii) sampling choices operated by Yip *et al.*[21].

### Evaluation procedure

In this work we compare our method to that of Yip *et al.*[21], where the authors evaluated their method using a 10-fold cross-validation procedure. To keep the comparison completely fair, we repeated said procedure with SBR, reusing the very same train/test splits. Since correlated objects, e.g. a protein and its domains/residues, share information, the folds were structured as to avoid such information to leak between train and test folds: this was achieved by keeping correlated objects in the same fold. In order not to bias the performance estimates, all redundant proteins were ignored, along with their domains and residues, when computing the results of both SBR and the method of Yip *et al.* The full experimental setup and instructions to replicate the experiments can be downloaded at http://sites.google.com/site/semanticbasedregularization/home/software/protein_interaction.

SBR has two scalar hyper-parameters that control the contribution of various parts of the objective function: *λ*_*c*_ is the weight associated to the constraints (how much the current solution is consistent with respect to the rules) and *λ*_*r*_, which controls the model complexity (see the Methods section for more details). The *λ*_*r*_ parameter was optimized on the first fold by training the model without the logic rules and it was then kept fixed for all the folds of the *k*-fold cross-validation. The resulting value is *λ*_*r*_=0.1. The *λ*_*c*_ parameter has not been optimized and kept fixed at *λ*_*c*_=1. Please note that further significant gains for the proposed method could be achieved by fine-tuning this meta-parameter. However, since the dataset from Yip *et al.* does not include a validation split, no sound way to optimize this parameter was possible without looking at the test set, or redefining the splits (making it difficult to compare against the results of Yip *et al.*). Therefore, we decided to not perform any tuning for this meta-parameter.

We computed three performance metrics: the Receiver Operating Characteristic (ROC) curve, the area under the ROC (AUCROC, or AUC for short), and the *F*_1_ score. The ROC curve represents the relation between the false positive rate (FPR) and the true positive rate (TPR), and can be seen as the proportion of true positives gained by “paying” a given proportion of false positives. By definition, the ROC curve is monotonically non-decreasing; the steeper the curve, the better the predictions. The AUC measures the ability to correctly discriminate between positives and negatives, or alternatively, the ability to rank positives above negatives. It is independent of any classification threshold, and thus particularly fit to evaluate models over the whole spectrum of possible decision thresholds. The *F*_1_ score is the harmonic mean of precision and recall. Contrary to the AUC, the *F*_1_ takes into account the predicted label, but not its confidence (margin).

**Figure 3:**
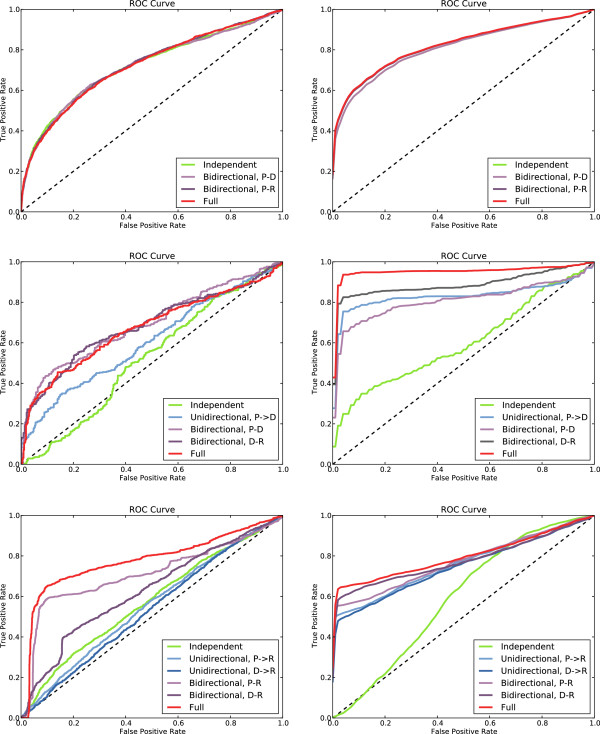
**ROC curves.** ROC curves obtained with the 10-fold cross-validation procedure, for all experimental settings and all levels of the hierarchy. (Left) Results for Yip *et al.* (Right) Results for SBR- ∃_*n*_. (Top) ROC curves for protein-level predictions with different sets of constraints, from fully independent to fully connected levels. (Middle) Domain-level predictions. (Bottom) Residue-level predictions. Each plot includes multiple ROC curves, one for each experimental setting; see the legends for more details.

We computed the average AUC and *F*_1_ of our method and those of our competitor over all folds of the cross-validation; the results can be found in Table 3 and Table 4. The ROC curves have been computed by collating the results of all test folds, and can be found in Figure 3. Since the ROC and *F*_1_ are not present in [21], and the dataset is slightly smaller because of the redundancy elimination step we introduced, we had to compute their results on a local re-run of their experiment. As a result, the AUC values presented in Table 3 are slightly different from those reported in [21]. However, we note that the results of our analysis would still apply if we had chosen to use the AUC values reported in [21].

**Table 3:** Results (AUC)

	**Independent**	**Unidirectional**			**Bidirectional**			**Full**
**Level**		**P → D**	**D → R**	**P → R**	**P ⇔ D**	**D ⇔ R**	**P ⇔ R**	
**Results for Yip *et al. *** [21]								
Proteins	0.723				0.722		0.725	0.724
Domains	0.531	0.619			0.688	0.695		0.673
Residues	0.563		0.542	0.549		0.576	0.659	0.722
**Results for SBR**								
Proteins	0.808				0.820		0.819	0.820
Domains	0.605	0.814			0.837	0.896		0.937
Residues	0.591		0.664	0.671		0.675	0.673	0.676
**Results for SBR-*∃***_***n***_								
Proteins	0.808				0.820		0.819	0.821
Domains	0.605	0.814			0.837	0.895		0.956
Residues	0.591		0.745	0.760		0.778	0.772	0.797

**Area under the ROC curve values attained by Yip *et al. *** [21]** , SBR, and SBR- ∃**_*n*_** (SBR equipped with the ***n***-existential quantifier).**

**Table 4:** **Results (*F***_**1**_**)**

	**Independent**	**Unidirectional**			**Bidirectional**			**Full**
**Level**		**P → D**	**D → R**	**P → R**	**P ⇔ D**	**D ⇔ R**	**P ⇔ R**	
**Results for Yip *et al. *** [21]								
Proteins	0.665				0.665		0.666	0.666
Domains	0.518	0.620			0.662	0.659		0.661
Residues	0.522		0.510	0.514		0.602	0.609	0.613
**Results for SBR**								
Proteins	0.718				0.722		0.722	0.723
Domains	0.568	0.693			0.696	0.731		0.750
Residues	0.579		0.605	0.605		0.605	0.605	0.602
**Results for SBR-*∃***_***n***_								
Proteins	0.717				0.722		0.722	0.722
Domains	0.568	0.693			0.696	0.729		0.757
Residues	0.579		0.635	0.639		0.641	0.644	0.650

***F***_**1**_** values attained by Yip *et al. *** [21]**, SBR, and SBR- ∃**_***n***_** (SBR equipped with the *n *-existential quantifier).**

### Results

To evaluate the effects of the constraints on the performances of SBR, we performed three independent experiments using rules of increasing complexity. This setup follows closely that of Yip *et al.*[21].

#### Independent levels

As a baseline, we estimate the performance of our method when constraints are ignored. This is equivalent to the method of Yip *et al.* when no information flow between levels is allowed. The results can be found in the “Independent” column of Tables 3 and 4.

In absence of constraints SBR reduces to standard *ℓ*_2_-regularized SVM classification: learning and inference become convex problems, and the method computes the globally optimal solution. Thus, the only differences between our method and the competitor are: (i) using classification versus regression, and (ii) using pairwise classification, instead of training a single model for each entity (protein, domain, residues). These differences alone produce a substantial increase in performance: the *F*_1_ changes by about +0.05 in all three cases. The AUC of proteins and domains is improved by about +0.09 and +0.07, respectively, while residues are less affected, with a +0.03 difference.

#### Unidirectional constraints

In the second experiment, we evaluate the effect of introducing unidirectional constraints between pairs of levels. In the P →D case only the P →D rule is active, meaning that bound protein pairs enforce positive domain pairs and negative domain pairs enforce negative protein pairs. The D →R and P →R cases are defined similarly. In all three cases, the level not appearing in the rule (e.g. the residue level in the P →D case) is predicted independently. This setup makes it easy to study the effects of propagating information from one level to the other without interferences. The results can be found in the “Unidirectional” column of Tables 3 and 4. In the same column we also show the results for Yip *et al.* for the unidirectional flow setting, where examples are propagated from one level to the next but not vice versa. However, since the competitor’s algorithm is iterative, information about lower levels can indeed affect the upper levels in successive iterations.

The results show that introducing unidirectional constraints in SBR improves the predictions in all cases. In particular, using (predicted and known) protein interactions helps inferring correct domain interactions, which improve by about +0.13 *F*_1_ and +0.2 AUC (P →D case). Residues improve independently of whether protein or domain-level information is used, with a +0.03 *F*_1_ in both cases, and a +0.08/ +0.07 AUC difference, respectively. Interestingly, proteins tend to help residue predictions slightly more than domains, despite the indirection between the two levels; this is likely an effect of the larger percentage of supervised pairs available.

Compared to SBR, the method of Yip *et al.* does not benefit as much from unidirectional information flow. Protein-level information allows to improve domain predictions only (+0.1 *F*_1_, +0.06 AUC for P →D), while residue predictions are worse than in the independent case (-0.05 and -0.04 AUC, and -0.01 *F*_1_, in the D →R and P →R cases, respectively).

#### Bidirectional constraints

In the third experiment we study the impact of using bidirectional constraints between pairs of levels; the level not appearing in the rules is predicted independently, as above. In the P ⇔D case, both the P →D and D →P rules are active, meaning that the protein and domain levels are enforced to be fully consistent; the P ⇔R and D ⇔R cases are defined analogously. This experiment is comparable to the bidirectional flow setting of Yip *et al.*. The results can be found in the “Bidirectional” column of Tables 3 and 4.

We observe that the new constraints have a positive effect on predictions at all three levels: proteins change from 0.808 AUC to 0.820, domains from 0.814 to 0.896 and residues from 0.671 to 0.673. In terms of *F*_1_, the changes are from 0.718 to (up to) 0.722 for proteins, from 0.693 to 0.731 for domains, and no change for residues. The change is not as marked as between the independent and unidirectional experiments. In particular, domains see the largest increase in performance (+0.08 AUC, +0.04 *F*_1_), in particular thanks to the contribution of residue-level information, which is more abundant. Proteins and residues are less affected. The result is unsurprising for proteins, which hold most of the supervision and are thus (i) more likely to be predicted correctly in the independent setting, and (ii) less likely to be assisted from hints coming from the other, less supervised levels.

As for the method of Yip *et al.*, the bidirectional flow mostly affects the domain and residue levels, whose improvement is +0.07 AUC/ +0.04 *F*_1_ and +0.11 AUC/ +0.09 *F*_1_, respectively; the change for protein interactions is negligible. Regardless of the relative performance increase, SBR is able to largely outperform the competitor in all configurations except one (*F*_1_ of the P ⇔R case for residues).

We note that the fact that all three cases (P ⇔D, P ⇔R and D ⇔R) improve over both the independent and the unidirectional experiments shows that not only the bidirectional constraints are in fact sound, but also that, despite the increased computational complexity, SBR is still able to exploit them appropriately.

#### All constraints

In the final experiment we activate the P →D, D →P, D →R and R →D rules, as defined in Table 2, making all levels interact. This is the most complex setting, and produces fully consistent predictions through the hierarchy. It is comparable to the “PDR” bidirectional setting of Yip *et al.*. The AUC scores can be found in column “Full” of Tables 3 and 4.

In this experiment the P →R and R →P constraints are not used. Direct information flow between proteins and residues is not needed, because it would be redundant: from a formal logic point of view, this corresponds to the observation that the logic rule expressing protein to residue consistency is implied by the other consistency rules. Indeed, we have experimentally verified that adding this propagation flow does not significantly affect the results.

In this experiment, protein predictions are stable with respect to the previous experiments, confirming the intuition that the abundance of supervision at this level makes it less likely to benefit from predictions at the other ones. On the contrary, domains see a large performance upgrade, from 0.896 to 0.937 AUC and from 0.731 to 0.750 *F*_1_, when made to interact with both proteins and residues. The change for residues is instead only marginal.

The results for Yip *et al.* are mixed, with proteins faring almost identically to the previous experiment, domains showing a slight drop in AUC but an equally slight increase in *F*_1_, and residues improving in AUC (+0.08) but not in *F*_1_ (unchanged) over the bidirectional P ⇔R case. The improvement in residue prediction (in terms of AUC) stands in contrast with the results of SBR, and is the only case in which the method of Yip and colleagues works better than SBR. The issue lies within our formulation of the D →R rule: whenever two domains are bound, the rule is satisfied when at least one residue pair is bound. As already mentioned above, this is not realistic: protein interfaces span more than two residues, typically five or more. We therefore extended SBR to support the *n*-existential quantifier, which allows to reformulate the D →R rule to take this observation into account (see the Methods section for more details on the *n*-existential quantifier). The new D →R rule, shown in Table 2, requires for each pair of bound domains at least *n*=5 residues to be bound. We chose the constant *n*=5 to be both realistic and, since the computational cost increases with *n*, small enough to be easily computable. We applied the same modification to the P →R rule.

**Figure 4:**
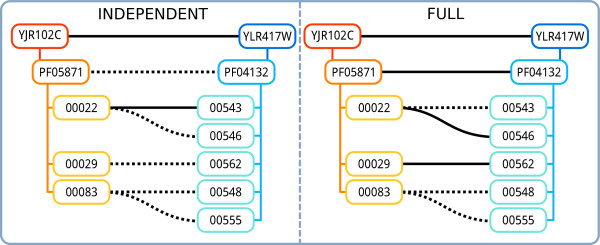
**Example prediction.** Prediction for the interaction between two ESCRT-II complex subunits: VPS25 (YJR102C) and VPS36 (YLR417W). The two proteins, their domains, and all the residue pairs in the dataset, are known to interact. Solid black lines indicate a predicted interaction, dotted lines a non-interaction; residue pairs not connected by either a solid or dotted line are not present in the dataset. (Left) SBR- ∃_*n*_ predictions with no constraints: the predictions at the three levels are inconsistent. (Right) SBR- ∃_*n*_ predictions with the full set of constraints: the protein- and domain-level predictions are now both consistent and correct. A further residue pair is now correctly predicted as interacting.

The complete results for the resulting method, termed SBR- ∃_*n*_, can be found at the bottom of Tables 3 and 4. When comparing to standard SBR, i.e., without the *n*-existential, we see that the performance of residues consistently improves in all cases (unidirectional, bidirectional, and with all constraints activated), allowing SBR- ∃_*n*_ to always outperform the method of Yip *et al.* by a significant margin also on the residue interactions. As a side effect of the better residue predictions, thanks to the D →R and R →D rules domains also improve in the all-constraints experiment. In particular, in the “Full” experiment the AUC improvement of SBR- ∃_*n*_ over Yip *et al.* is +0.1/ +0.26/ +0.07 AUC and +0.06/ +0.1/ +0.04 *F*_1_ for proteins, domains and residues respectively. We show in Figure 4 an example prediction obtained by SBR- ∃_*n*_ for the VPS25 and VPS36 ESCRT-II complex subunits. The figure shows that, while the unconstrained (baseline) predictions are inconsistent, the addition of the constraints effectively makes the protein- and domain-level predictions correct and consistent, and enables SBR to improve the residue-level predictions.

Summing up, these results highlight the ability of SBR to enforce constraints even with highly complex combinations of rules, allowing the modeler to fully exploit the flexibility and performance improvement offered by non-standard FOL extensions like the *n*-existential operator.

### Discussion

The results presented in the previous section offer a clear perspective on the advantages of the proposed method. By employing appropriate classification techniques and training a single global pairwise model per level, rather than relying on the less than optimal local (per-object) regression models of Yip *et al.*, a considerable improvement was achieved even in the unconstrained experiment. Furthermore, when enforcing consistency among the protein, domain and residue levels and using the *n*-existential quantifier, the experimental results are significantly better than both the unconstrained baseline and the corresponding results of Yip and colleagues, at all levels and in all experimental settings.

It is worth noting that SBR performance improves monotonically with the increase of constraint complexity in the reported experiments. This result is far from obvious, and confirms both that the biologically-motivated knowledge base is useful, and that SBR is able to effectively apply it. In contrast, the competitor’s method does not always improve in a similar manner.

In general, the performance gain brought forth by inter-level propagation is not homogeneously distributed between the three levels. We register a large improvement for domains and residues, especially when SBR is used in conjunction with the *n*-existential quantifier. Proteins are less affected by consistency enforcement, most likely due to the availability of more supervised examples.

We note that the FOL rules have a twofold effect. Firstly, they propagate information between the levels, enabling predicted interactions at one level to help inferring correct interactions at the other two levels. This is especially clear in the “Full” experiment with the *n*-existential quantifier: in this case, better residue level predictions increase the overall quality of domain predictions as well. Secondly, the rules also guarantee that the predictions are consistent along the object hierarchy.

Summarizing, SBR is able to largely outperform that of Yip and colleagues, and moreover enforces the predictions to be consistent among levels. As previously mentioned, the data taken from Yip *et al.* has some peculiarities worth discussing. First, it contains a low number of residue–residue interactions, partially due to design choices taken by [21]. Second, it is artificially balanced by including an appropriate number of non-interactions, while in a real-world case all possible pairs would qualify as candidates. We decided to keep the dataset as-is in order to facilitate a fair comparison with Yip *et al.*. We postpone further analysis with other datasets to future work.

## Conclusions

In this work we tackle the multi-level protein interaction prediction (MLPIP) problem, first introduced by Yip *et al.*[21], which requires to establish the binding state of all uncharacterized pairs of proteins, domains and residues. Contrary to standard protein–protein interaction prediction, the MLPIP problem offers many advantages and opens up new challenges. The primary contribution of this paper is the extension and application to the MLPIP task of a state-of-the-art statistical relational learning technique called Semantic Based Regularization.

SBR is a flexible framework to inject domain knowledge into kernel machines. In this paper SBR has been used to tie together protein, domain and residue interaction predictions tasks. In particular, the domain knowledge expresses that two proteins interact if and only if there is an interaction between at least one pair of domains of the proteins. Similarly two domains can interact if and only if there are at least some residues interacting. While these tasks could be learned separately, tying them together has multiple advantages. First the predictions will be consistent and more accurate, as the predictions at one level will help the predictions at the other levels. Secondly, the domain knowledge can be enforced also on the unsupervised data (proteins, domains and residues for which interactions are unknown). Unsupervised data is typically abundant in protein interaction prediction tasks but often neglected. This methodology allows to powerfully leverage it, significantly improving the prediction accuracy. Note also that, while the resolved complexes are required during the training stage, no structural information is required for performing inference on novel proteins.

While other work in the literature has exploited the possibility of tying the predictions at multiple levels, the presented methodology employs a more principled inference process among the levels, where the domain knowledge can be exactly represented and precisely enforced. The experimental results confirm the theoretical advantages by showing significant improvements in domain and residue interaction prediction accuracy both with respect to approaches performing independent predictions and the only previous approach attempting at linking the prediction tasks.

Given the flexibility offered by SBR, the proposed method can be extended in several ways. The simplest extension involves engineering a more refined rule set, for instance by introducing (soft) constraints between the binding state of consecutive residues, which are likely to share the same state. More ambitious goals, requiring a redesign of the experimental dataset, include encoding selected information sources, such as domain types, subcellular co-localization and Gene Ontology annotations, as First Order Logic constraints rather than with kernels, to better leverage their relational nature.

## Methods

### Kernel machines

Machine learning and statistical methods are very well defined for the linear case, and statistical learning theory can provide optimal solutions in terms of generalization performance. Unfortunately, non-linearity is often required in order to solve most applications, where exploiting complex dependencies is essential to predict some higher level property of the objects. Kernel methods try to combine the potential classification power of non-linear methods and the optimality and computational efficiency of linear methods by mapping the input patterns into a high dimensional feature space, where parameter optimization remains linear.

Kernel methods have a wide range of applications in many fields, and can be used for many different tasks like regression, clustering and classification (the latter being the main interest of this paper). In particular, the representer Theorem [46] shows that a large class of problems admits solutions in terms of kernel expansions having the following form:

(2) $$f(x)=\overset{N}{\underset{i=1}{\sum }}w_{i}K(x,x_{i})$$

where ***x*** is the representation of the pattern, *K*(***x***, ***x***_*i*_)=<*Φ*(***x***),*Φ*(***x***_*i*_)> is a kernel function, where *Φ*(·) is some mapping from the input space to the feature space. Intuitively, the kernel function measures the similarity between pairs of instances, and the prediction *f*(***x***) for a novel instance is computed as a weighted similarity to training instances ***x***_*i*_. There is a large body of literature on kernel machines, see e.g. [26] for an introduction.

The optimization of the weights *w*_*i*_ of the Kernel machine can be formulated in different ways. Let us indicate *y*_*j*_∈{-1,+1} the desired output for pattern ***x***_*i*_, ***w*** = [ *w*_1_,…,*w*_*n*_] is a vector arranging the kernel machine parameters and ***G*** is the gram matrix, having its (*i*,*j*) element defined as ***G***_*i* *j*_=*K*(***x***_*i*_, ***x***_*j*_). ||*f*||^2^= ***w***^*t*^ ***G*** ***w***, and it can be shown that the following cost function:

$$\left||f^{2}||+\lambda \overset{N}{\underset{j=1}{\sum }}L(y_{j},f(x_{j})\right)$$

reduces to the formulation of hard margin *ℓ*_2_SVMs [23] if *L*(·) is the hinge loss and *λ*→*∞*. A very similar cost function has been employed to solve the protein, domain, residue interaction presented in this paper.

### First-order logic

Propositional logic is based on the basic concept of propositions, which can assume either a true or false value. It is possible to perform operations on the propositions by connecting them via the *and* (∧), *or* (∨) and *not* (¬) operators. In particular, given two propositions *A*,*B*, it holds that: *A*∧*B* is true iff *A*=*true*,*B*=*true*, *A*∨*B* is false iff *A*=*false*,*B*=*false* and ¬*A* flips the current truth value of *A*. The operator ⇒ can be used to express a conditional statement: *A*⇒*B* expresses the fact that B must hold true if *A* is true. The sentence *A*⇒*B* is false iff *A*=*true* and *B*=*false*, and it can be expressed in terms of other operators through the equivalence *A*⇒*B*≡¬*A*∨*B*.

First-order logic (FOL) extends propositional logic to compactly express generic properties for a class of objects, thanks to the use of predicates, variables and quantifiers. A *variable* can assume as value any object in some considered domain. A variable is said to be *grounded* once it is assigned a specific object. A *predicate* is a function that, taking as input some objects (or grounded variables), returns either true or false. Predicates can be connected with other predicates using the same operators defined for propositional logic. The *universal quantifier* (∀) expresses the fact that some proposition is true for any object, while the *existential quantifier* (∃) expresses the fact that some proposition is true for at least one object.

For example, let x be a variable and let Protein(x),Enzyme(x),NonEnzyme(x) indicate three predicates expressing whether, given a grounding x=PDB1a3a, PDB1a3a is a protein, an enzyme, a non-enzyme, respectively. The following FOL clause can be used to express that any protein is either an enzyme or it is not: ∀x Protein(x)⇒ Enzyme(x)∨NonEnzyme(x).

Variables and quantifiers can be combined. For example, given the predicates Protein(x) holding true if x is a protein and ResidueOf(x, y) holding true if y is a residue of x, the following clause expresses the fact that any protein has a at least one residue: ∀x Protein(x)⇒∃y ResidueOf(x,y).

### Semantic-based regularization

Semantic Based Regularization (SBR) [22] is a general framework for injecting prior knowledge expressed in FOL into kernel machines for semi-supervised learning tasks. The prior knowledge is converted into a set of continuous constraints, which are enforced during training. The SBR framework is very general and allows to employ the full expressiveness of FOL in the definition of the prior knowledge. The SBR framework also allows to perform collective classification on the test set, in order to enforce the output to respect the logic knowledge.

Let us consider a multitask learning problem, where each task works on an input domain where labeled and unlabeled examples are sampled from. For example, in the case study presented in this paper, three separate tasks for protein, domain and residue interaction need to be conjunctively learned. Each input pattern is described via some representation that is relevant to solve the tasks at hand. Let us indicate with *T* the total number of tasks, where task *k* is implemented by a function *f*_*k*_, which lives in an appropriate Reproducing Kernel Hilbert Space. In the following, ***f*** = [*f*_1_,…,*f*_*T*_]^′^ indicates the vector collecting all task functions. The basic assumption of SBR is that the task functions are correlated as they have to meet a set of constraints that can be expressed by the functionals $\phi_{h}:{ℋ}_{1}\times \ldots \times {ℋ}_{T}\to [0,+\infty )$ such that *ϕ*_*h*_(***f***)=0 *h*=1,…,*H* must hold for any valid choice of $f_{k}\in {ℋ}_{k},k=1,\ldots ,T$. Following the classical penalty approach for constrained optimization, the constraints are embedded by adding a term that penalizes their violation:

(3) $$\begin{array}{ll} \lambda_{r}\overset{T}{\underset{k=1}{\sum }}||f_{k}|{|}^{2} & +\overset{T}{\underset{k=1}{\sum }}\underset{\left(x_{k}^{j},y_{k}^{j}\right)\in {ℒ}_{k}}{\sum }\;\;\;L\left(f_{k}(x_{k}^{j}),y_{k}^{j}\right)+\\ & +\lambda_{c}\overset{H}{\underset{h=1}{\sum }}\phi_{h}(S,f), \end{array}$$

where *L*(·) is a loss function measuring the distance of the function output from the desired one and  is the considered sample of data points over which the functions are evaluated. In the experimental setting, *L*(·) has been set to be the hinge function. It is possible to extend the Representer Theorem to show that the best solution for Equation 3 can be expressed as a kernel expansion as showed in Equation 2 [22].

Therefore, using kernel expansions, Equation 3 becomes:

$$\begin{array}{ll} \lambda_{r}\overset{T}{\underset{k=1}{\sum }}w_{k}^{\prime }G_{k}w_{k} & +\overset{T}{\underset{k=1}{\sum }}L\left(G_{k}w_{k},y_{k}\right)+\\ & +\lambda_{c}\overset{H}{\underset{h=1}{\sum }}\phi_{h}(G_{1}w_{1},\ldots ,G_{T}w_{T}), \end{array}$$

where ***G***_*k*_, ***w***_*k*_, ***f***_*k*_= ***G***_*k*_ ***w***_*k*_ and ***y***_*k*_ are the gram matrix, the weights, the function values over the data sample and the desired output column vectors for the patterns in the domain of the *k*-th task. Evidence tasks do not need to be approximated as their are fully known.

Optimization of the ***w***_*k*_ parameters for the cost function in Equation 4 can be done using gradient descent. Constraint *ϕ*_*h*_ are non-linear in most interesting cases like the one presented in this paper. Therefore, the cost function can present multiple local minima, making optimization difficult. SBR uses a two-step heuristic to solve this problem: first it computes the theoretically global optimum for all predicates independently (setting *λ*_*c*_=0), which are convex kernel machines. Then, it introduces the constraints and proceeds to find a good solution using a gradient descent.

In the following we show how to express first order logic clauses in terms of constraints *ϕ*_*h*_.

### Translation of first-order logic clauses into real-valued constraints

With no loss of generality, we restrict our attention to FOL clauses in the PNF form, where all the quantifiers (∀,∃) and their associated quantified variables are placed at the beginning of the clause. For example:

(4) $$\overset{\text{Quantifiers}}{\overset{︷}{\forall v_{1}\forall_{v_{2}}}}\overset{\text{Quantifier}-\text{free}\;\text{expression}}{\overset{︷}{A(v_{1})\wedge B(v_{2})\Rightarrow C(v_{1})}}$$

Please note that the quantifier-free part of the expression is equivalent to an assertion in propositional logic for any given grounding of the quantified variables. As studied in the context of fuzzy logic and symbolic AI, different methods can be used for the conversion of a propositional expression into a continuous function with [ 0,1] input variables.

#### T-norms

T-norms [47] are commonly used in fuzzy logic [48] to generalize propositional logic expressions to real valued functions of continuos variables. A continuous t-norm is a function t:[0,1]×[0,1]→R, that is continuous, commutative, associative, monotonic, and featuring a neutral element 1 (i.e. *t*(*a*,1)=*a*). A *t-norm fuzzy logic* is defined by its t-norm *t*(*a*_1_,*a*_2_) that models the logic AND, while the negation of a variable ¬*a* is computed as 1-*a*. Once defined the t-norm functions corresponding to the logical AND and NOT, these functions can be composed to convert any arbitrary logic proposition into a continuous function. Many different t-norm logics have been proposed in the literature. For example, the *product t-norm* used in the experimental section:

$$\begin{array}{lll} \left(a_{1}\wedge a_{2}\right) & \overset{\text{mapped}}{\to } & t(a_{1},a_{2})=a_{1}\cdot a_{2}\\ \left(\neg a_{1}\right) & \overset{\text{mapped}}{\to } & t(a_{1})=1-a_{1}\\ \left(a_{1}\vee a_{2}\right) & \overset{\text{mapped}}{\to } & t(a_{1},a_{2})=a_{1}+a_{2}-a_{1}\cdot a_{2} \end{array}$$

Please note that the t-norm behaves as classical logic when the variable approaches the value 0 (false) or 1 (true).

The equivalence *a*_1_⇒*a*_2_≡¬*a*_1_∨*a*_2_ can be used to represent implications (*modus ponens*) before performing t-norm conversion. However, this process does not powerfully capture the inference process performed in a probabilistic or fuzzy logic context. Any t-norm has a corresponding binary operator ⇒ called *residuum*, which is used in fuzzy logic to generalize implications in case of continuous variables. In particular, for a minimum t-norm, it holds that the residuum converting an implication is defined as:

$$(a_{1}\Rightarrow a_{2})\;\;\overset{\text{mapped}}{\to }\;\;t(a_{1},a_{2})=\left\{\begin{array}{lll} 1 & \;\;\; & a_{1}\leq a_{2}\\ a_{2} & \;\;\; & a_{1}>a_{2} \end{array}\right)$$

The residuum allows to relax the condition of satisfaction for the implication: an implication is satisfied if the right end side of the implication is more verified than the pre-condition on the left side. This makes the fuzzy or probabilistic inference process easier and better defined. While the original SBR formulation represents implications using modus ponens, the minimum t-norm *residuum* has been used in the experimental section of this paper to convert implications.

For example, the quantifier-free expression in Equation 4 corresponds to:

(5) $$\left\{\begin{array}{lll} 1 & \;\;\; & f_{A}(x_{1})\cdot f_{B}(x_{2})\leq f_{C}(x_{1})\\ f_{B}(x_{2}) & \;\;\; & \text{else} \end{array}\right)$$

where the predicates have been substituted by the unknown corresponding functions and ***x***_1_, ***x***_2_ are the representations of the objects identified by the grounded variables *v*_1_,*v*_2_, respectively. The representation of the object must be compatible with what is accepted as input by the kernels used by the predicate approximations *f*_*A*_,*f*_*B*_. For example, they can be a vector of real valued features when using a linear or Gaussian kernel, or graph or tree representations when using kernel for structures. It is also possible to use the methodology when no explicit representations are known, but only the kernel values for each pair of input objects.

Quantifiers are also converted into real value operators. The universal quantifier corresponds to the sum of the degrees of violation of the continuous expression coming from t-norms over all possible groundings for the quantified variable. Let us consider a universally quantified FOL formula ∀vE(v,P). When considering the real–valued mapping *t*_*E*_(***f***, ***x***) of the original boolean expression where ***x*** is the representation of *v*, the universal quantifier can be converted measuring the degree of non-satisfaction of the expression over the domain  of ***x***:

$$\forall v\;\;E(v,P)\;\;\overset{\text{mapped}}{\to }\;\;\phi (f,S)=\underset{x\in S}{\sum }1-t_{E}(f,x)$$

For example, the formula ∀*vA*(*v*)∧*B*(*v*) corresponds to:

$$\phi (f,S)=\underset{x\in S}{\sum }1-f_{A}(x)f_{B}(x)$$

where *f*_*A*_(***x***)*f*_*B*_(***x***) is the t-norm generalization of the propositional expression *A*(*v*)∧*B*(*v*) for a given grounding of *v*.

When multiple universally quantified variables are present, the conversion is performed recursively from the outer to the inner variable. In particular, $\forall v_{1}\ldots \forall v_{n}\;E(P,v_{1},\ldots ,v_{n})$ is mapped to the constraint:

$$\phi (f,S)=\underset{x_{1}\in S_{1}}{\sum }\ldots \underset{x_{n}\in S_{n}}{\sum }1-t_{E}(f,x_{1},\ldots ,x_{n})$$

For example, the formula ∀*v*_1_∀*v*_2_*A*(*v*_1_)∧¬*B*(*v*_2_) corresponds to:

$$\phi (f,S)=\underset{x_{1}\in S_{1}}{\sum }\underset{x_{2}\in S_{2}}{\sum }1-f_{A}(x_{1})(1-f_{B}(x_{2}))$$

The existential quantifier is mapped into the continuos domain as:

$$\exists v\;E(P,v)\;\;\overset{\text{mapped}}{\to }\;\;\phi (f,S)=\underset{x\in S}{\min}1-t_{E}(f,x)$$

This framework also allows a natural definition of the ∃_*n*_ operator, generalizing the existential operator to *n* objects. This operator is usually defined in description logic, while it can only indirectly defined in FOL. This operator will be used in the experimental section and its continuous mapping is defined as:

$$\exists_{n}v\;\;E(v,P)\;\;\overset{\text{mapped}}{\to }\;\;\phi (f,S)=\underset{{\arg\max}_{n}x\in \mathrm{Sn}}{\sum }1-t_{E}(f,x)$$

where ${\text{argmax}}_{n}x\in \mathrm{Sn}$ indicates the *n* assignments of ***x*** that maximize the value of 1-*t*_*E*_(·) over the set . The conversion of the ∃_*n*_ operator consistently reduces to the ∀ conversion when n=|S|, and to the conversion of the ∃ operator when *n*=1.

As a final example of the conversion procedure, let’s refer to the FOL clause in Equation 4, which is converted into the real-valued constraint ϕ(f,S):

$$\underset{x_{1}\in S_{1}}{\sum }\underset{x_{2}\in S_{2}}{\sum }\;\left\{\;\;\begin{array}{ll} 0 & f_{A}(x_{1})\cdot f_{B}(x_{2})\leq f_{C}(x_{1})\\ 1\;-\;f_{B}(x_{2}) & \text{else} \end{array}\right)$$

## Competing interests

The authors declare that they have no competing interests.

## Authors’ contributions

CS and ST implemented and carried out the experiments. MD and AP provided the original idea and supervised the experiments. All authors wrote and approved the final manuscript.

## References

[B1] Keskin O Gursoy A Ma B Nussinov R **Principles of protein-protein interactions: what are the preferred ways for proteins to interact?** Chem Rev 2008 108 4 1225 1244 18355092 10.1021/cr040409x

[B2] Hopkins AL **Network pharmacology: the next paradigm in drug discovery** Nat Chem Biol 2008 4 11 682 690 18936753 10.1038/nchembio.118

[B3] Csermely P Korcsmáros T Kiss HJ London G Nussinov R **Structure and dynamics of molecular networks: A novel paradigm of drug discovery** Pharmacol Ther 2013 138 3 333 408 23384594 10.1016/j.pharmthera.2013.01.016 PMC3647006

[B4] Tuncbag N Kar G Keskin O Gursoy A Nussinov R **A survey of available tools and web servers for analysis of protein–protein interactions and interfaces** Brief Bioinform 2009 10 3 217 232 19240123 10.1093/bib/bbp001 PMC2671387

[B5] Lewis AC Saeed R Deane CM **Predicting protein–protein interactions in the context of protein evolution** Mol Biosyst 2010 6 55 64 20024067 10.1039/b916371a

[B6] Skrabanek L Saini HK Bader GD Enright AJ **Computational prediction of protein–protein interactions** Mol Biotechnol 2008 38 1 17 18095187 10.1007/s12033-007-0069-2

[B7] Wass MN David A Sternberg MJ **Challenges for the prediction of macromolecular interactions** Curr Opin Struct Biol 2011 21 3 382 390 21497504 10.1016/j.sbi.2011.03.013

[B8] Cary MP Bader GD Sander C **Pathway information for systems biology** FEBS Lett 2005 579 8 1815 1820 15763557 10.1016/j.febslet.2005.02.005

[B9] Shoemaker BA Panchenko AR **Deciphering protein–protein interactions. Part II. Computational methods to predict protein and domain interaction partners** PLoS Comput Biol 2007 3 4 e43 17465672 10.1371/journal.pcbi.0030043 PMC1857810

[B10] Ezkurdia I Bartoli L Fariselli P Casadio R Valencia A Tress ML **Progress and challenges in predicting protein–protein interaction sites** Brief Bioinform 2009 10 3 233 246 19346321 10.1093/bib/bbp021

[B11] Fields S Song O **A novel genetic system to detect protein protein interactions** Nature 1989 340 6230 245 246 2547163 10.1038/340245a0

[B12] Shoemaker BA Panchenko AR **Deciphering protein–protein interactions. Part I. experimental techniques and databases** PLoS Comput Biol 2007 3 3 e42 17397251 10.1371/journal.pcbi.0030042 PMC1847991

[B13] Memiševič V Wallqvist A Reifman J **Reconstituting protein interaction networks using parameter-dependent domain-domain interactions** BMC Bioinformatics 2013 14 154 23651452 10.1186/1471-2105-14-154 PMC3660195

[B14] Radivojac P Clark WT Oron TR Schnoes AM Wittkop T Sokolov A Graim K Funk C Verspoor K Ben-Hur A Pandey G Yunes JM Talwalkar AS Repo S Souza ML Piovesan D Casadio R Wang Z Cheng J Fang H Gough J Koskinen P Törönen P Nokso-Koivisto J Holm L Cozzetto D Buchan DW Bryson K Jones DT Limaye B **A large-scale evaluation of computational protein function prediction** Nat Methods 2013 10 3 221 227 23353650 10.1038/nmeth.2340 PMC3584181

[B15] Jiang JQ Wu M **Predicting multiplex subcellular localization of proteins using protein-protein interaction network: a comparative study** BMC Bioinformatics 2012 13 Suppl 10 S20 22759426 10.1186/1471-2105-13-S10-S20 PMC3314587

[B16] Zhao XM Wang RS Chen L Aihara K **Uncovering signal transduction networks from high-throughput data by integer linear programming** Nucleic Acids Res 2008 36 9 e48 e48 18411207 10.1093/nar/gkn145 PMC2396433

[B17] Nguyen TP Ho TB **Discovering signal transduction networks using signaling domain-domain interactions** Genome Inform 2006 17 2 35 45 17503377

[B18] Nguyen TP Ho TB **An integrative domain-based approach to predicting protein–protein interactions** J Bioinform Comput Biol 2008 6 06 1115 1132 19090020 10.1142/s0219720008003874

[B19] Pitkänen E Rousu J Ukkonen E **Computational methods for metabolic reconstruction** Curr Opin Biotechnol 2010 21 70 77 20171871 10.1016/j.copbio.2010.01.010

[B20] Tuncbag N Gursoy A Keskin O **Identification of computational hot spots in protein interfaces: combining solvent accessibility and inter-residue potentials improves the accuracy** Bioinformatics 2009 25 12 1513 1520 19357097 10.1093/bioinformatics/btp240

[B21] Yip KY Kim PM McDermott D Gerstein M **Multi-level learning: improving the prediction of protein, domain and residue interactions by allowing information flow between levels** BMC Bioinformatics 2009 10 241 19656385 10.1186/1471-2105-10-241 PMC2734556

[B22] Diligenti M Gori M Maggini M Rigutini L **Bridging logic and kernel machines** Mach Learn 2012 86 57 88

[B23] Cortes C Vapnik V **Support-vector networks** Mach Learn 1995 20 3 273 297

[B24] Getoor L Taskar B Introduction to Statistical Relational Learning 2007

[B25] Raedt LD, Frasconi P, Kersting K, Muggleton S Probabilistic Inductive Logic Programming - Theory and Applications, Volume 4911 of Lecture Notes in Computer Science 2008

[B26] Hofmann T Schölkopf B Smola AJ **Kernel methods in machine learning** Ann Stat 2008 36 3 1031 1508

[B27] Ben-Hur A Noble WS **Kernel methods for predicting protein–protein interactions** Bioinformatics 2005 21 suppl 1 i38 i46 15961482 10.1093/bioinformatics/bti1016

[B28] Chapelle O Schölkopf B Zien A Semi-Supervised Learning, Volume 2 2006 MIT press

[B29] Zhu X **Semi-supervised learning literature survey** Computer Science, University of Wisconsin-Madison 2006 2 3 [http://pages.cs.wisc.edu/jerryzhu/research/ssl/semireview.html]

[B30] Hwang H Pierce B Mintseris J Janin J Weng Z **Protein–protein docking benchmark version 3.0** Proteins: Struct, Funct, Bioinf 2008 73 3 705 709 10.1002/prot.22106 PMC2726780 18491384

[B31] Li B Kihara D **Protein docking prediction using predicted protein-protein interface** BMC Bioinformatics 2012 13 7 22233443 10.1186/1471-2105-13-7 PMC3287255

[B32] Qi Y Noble WS **Protein interaction networks: protein domain interaction and protein function prediction** Handbook of Statistical Bioinformatics 2011 427 459

[B33] Bock JR Gough DA **Predicting protein–protein interactions from primary structure** Bioinformatics 2001 17 5 455 460 11331240 10.1093/bioinformatics/17.5.455

[B34] Qi Y Tastan O Carbonell JG Klein-Seetharaman J Weston J **Semi-supervised multi-task learning for predicting interactions between HIV-1 and human proteins** Bioinformatics 2010 26 18 i645 i652 20823334 10.1093/bioinformatics/btq394 PMC2935441

[B35] You ZH Lei YK Gui J Huang DS Zhou X **Using manifold embedding for assessing and predicting protein interactions from high-throughput experimental data** Bioinformatics 2010 26 21 2744 2751 20817744 10.1093/bioinformatics/btq510 PMC3025743

[B36] Xia Z Wu LY Zhou X Wong ST **Semi-supervised drug-protein interaction prediction from heterogeneous biological spaces** BMC Syst Biol 2010 4 Suppl 2 S6 20840733 10.1186/1752-0509-4-S2-S6 PMC2982693

[B37] Nguyen TP Ho TB **Detecting disease genes based on semi-supervised learning and protein–protein interaction networks** Artif Intell Med 2012 54 63 71 22000346 10.1016/j.artmed.2011.09.003

[B38] De Raedt L Inductive Logic Programming 2010

[B39] Tran TN Satou K Ho TB **Using inductive logic programming for predicting protein-protein interactions from multiple genomic data** Knowledge Discovery in Databases: PKDD 2005 2005 321 330

[B40] González AJ Liao L Wu CH **Prediction of contact matrix for protein–protein interaction** Bioinformatics 2013 29 8 1018 1025 23418186 10.1093/bioinformatics/btt076 PMC3624801

[B41] Smola AJ Schölkopf B **A tutorial on support vector regression** Stat Comput 2004 14 3 199 222

[B42] Blum A Mitchell T **Combining labeled and unlabeled data with co-training** Proceedings of the Eleventh Annual Conference on Computational Learning Theory 1998 ACM 92 100

[B43] Altschul SF Madden TL Schäffer AA Zhang J Zhang Z Miller W Lipman DJ **Gapped BLAST and PSI-BLAST: a new generation of protein database search programs** Nucleic Acids Res 1997 25 17 3389 3402 9254694 10.1093/nar/25.17.3389 PMC146917

[B44] Gomez SM Noble WS Rzhetsky A **Learning to predict protein–protein interactions from protein sequences** Bioinformatics 2003 19 15 1875 1881 14555619 10.1093/bioinformatics/btg352

[B45] Li W Godzik A **Cd-hit: a fast program for clustering and comparing large sets of protein or nucleotide sequences** Bioinformatics 2006 22 13 1658 1659 16731699 10.1093/bioinformatics/btl158

[B46] Schölkopf B Smola AJ Learning with Kernels 2002

[B47] Klement E Pap E Mesiar R Triangular Norms 2000

[B48] Klir G Yuan B Fuzzy Sets and Fuzzy Logic: Theory and Applications 1995

